# Immunonutrition within enhanced recovery after surgery (ERAS): an unresolved matter

**DOI:** 10.1186/s13741-017-0080-5

**Published:** 2017-12-11

**Authors:** Ruchir Gupta, Anthony Senagore

**Affiliations:** 1 Department of Anesthesiology, Stony Brook School of Medicine, 101 Nicholls Drive, Stony Brook, NY USA; 2 University of Texas Medical Branch, 301 University Boulevard, Galveston, TX 77555-0541 USA

## Abstract

Preoperative malnutrition because of poor oral intake significantly increases the risk of adverse events after surgery and leads to increased length of stay. While immunonutrition has been utilized in the non-ERAS setting, its utility in both minimally invasive surgery and ERAS pathway procedures remain poorly defined. There are at least ten meta-analyses regarding the assessment of immunonutrition, but virtually, all of these were performed in an era prior to minimally invasive surgery, adoption of enhanced recovery protocols, and an understanding of the assessment and physiology of sarcopenia. In terms of immunonutrition within an ERAS pathway, the few studies that have been published have severe flaws in design and sample, bringing their overall conclusion into question. Furthermore, the optimal components of immunonutrition have yet to be adequately determined and may vary for patients based on comorbidities as well as the proposed procedures. Risk stratification based on markers of nutritionally deficient states such as image assessed sarcopenia, Glasgow Prognostic Score, prognostic nutrition index, or assessment of methylarginines are needed prior to the initiation of any such immunotherapy. Lastly, there is a need for properly designed randomized control trials that stratify patients appropriately and determine the optimal timing, composition, and duration of immunotherapy.

In recent years, several standard nutrition preparations have been modified by adding specific nutrients, such as arginine, ω-3 fatty acids, glutamine, and others. Though these substances, termed “immunonutrition,” have been shown to upregulate host immune response, modulate inflammatory response, and improve protein synthesis after surgery, their role within an enhanced recovery pathway remains unclear. Furthermore, though there is clear evidence that sarcopenia, malnutrition, arginine bioavailability, and a high arginase activity state in isolation or in combination contribute to adverse surgical outcomes, the precise combination of these elements remains unclear and is in evolution.

## Traditional use of immunonutrition

There are at least ten meta-analyses regarding the assessment of immunonutrition. However, virtually all of these were performed in an era prior to minimally invasive surgery, adoption of enhanced recovery protocols, and an understanding of the assessment and physiology of sarcopenia. A meta-analysis by Braga (2013) strongly favored the use of immunonutrition in high risk elective surgical patients. However, as the authors themselves admitted, many of the included studies were done over 2 decades prior to their review and thus did not reflect the value of immunonutrition within the context of recent advances.

Conversely, a meta-analysis performed by Hegazi (2014) assessed 561 patients across eight randomized controlled trials (RCTs) of preoperative immunonutrition vs. standard enteral therapy, as well as nine RCTs of immunonutrition vs. no supplements. Meta-analysis was performed for reported outcomes including wound infection, infectious and non-infectious complications, and length of stay (LOS). During their analysis, the authors were unable to determine a benefit of immunonutrition compared to standard enteral nutritional support. Thus, the authors concluded that standard enteral nutrition offers an alternative to immunonutrition for preoperative nutritional supplementation.

Thus, the results of neither meta-analyses have significantly advanced the science of immunonutrition.

## Optimal nutrient mix of immunonutrition

The assumption that the nutritional needs and catabolic state are the same for the various clinical scenarios is a major gap in current knowledge. For example, a patient undergoing neoadjuvant chemoradiation followed by a Whipple procedure for a pancreatic neoplasm is very different from an elective laparoscopic colectomy.

Thus, the actual composition of the immunonutrition may vary from patient group to patient group. Interestingly, most of the recommended nutritional formulas contain arginine, omega-3 fatty acids, carbohydrate/protein ratios, and glutamine all of which have been associated with neutral to negative outcomes in large randomized trials in critically ill patients.

For example, Martin et al. (2010) reviewed the existing data on the role of omega-3 fatty acids in critical illness and found that the data is often contradictory and inconclusive. The authors concluded that supplementation with omega-3 fatty acids may influence the acute inflammatory response in critically ill patients, but further research into this subject is required before any definitive recommendations could be made.

Similarly, Andrews and colleagues (2011) reached a similar conclusion in their study when assessing the added benefit of selenium and glutamine. The authors concluded that while the inclusion of selenium (500 mcg for 5 days) in a standard isonitrogenous, isocaloric preparation of parenteral nutrition may reduce the onset of new infections, there was no evidence of benefit with glutamine supplementation. A similar conclusion was reached by Heyland and colleagues (2013) in which glutamine supplementation was studied in critically ill patients. The authors found that not only did early provision of glutamine not improve clinical outcomes, but also that glutamine was associated with an increase in mortality with multiorgan failure. The controversy regarding the potential benefit related to enteral glutamine may be related to its conversion to citrulline which is subsequently absorbed and converted to arginine in the kidney.

With respect to arginine, a review by Dioguardi et al. (2011) concluded that exogenous arginine supplementation should be avoided because it does not resolve the reasons underlying its excess consumption or blunted synthesis, but rather may actually worsen them. Instead, balanced formulations of essential AA may be of benefit because these would better maintain and restore concentrations and metabolic pathways for the synthesis of arginine.

## Proper stratification of diverse populations

A further challenge of the extant literature is that the majority of assessed studies had small sample size (< 100), and no current study has used current markers of nutritionally impaired states (i.e., image assessed sarcopenia, Glasgow Prognostic Score, prognostic nutrition index, or assessment of methylarginines) when assessing outcomes.

Traditionally, patients have been screened or assessed for either malnutrition or sarcopenia, but rarely for both conditions even though many patients present clinically.

The various populations of sarcopenic, sarcopenic/malnourished, and malnourished should be assessed with respect to optimal nutrient mix and duration of repletion perioperatively. Vandewoude and colleagues (2012) have recently coined the term “malnutrition-sarcopenia syndrome (MSS).” The European Working Group on Sarcopenia in Older People (EWGSOP) defines sarcopenia as an age-related loss of muscle mass, combined with the loss of strength, functionality, or both (Cruz-Jentoft et al. 2010). Sarcopenia as a condition is a major cause of frailty and disability in older adults; as an active process, it is present in every person reaching adult life. The authors have suggested that patients should be screened for not just malnutrition (which is a well-known cause of morbidity and mortality) but also for sarcopenia as both are commonly co-existing conditions, especially in older adults. Both these conditions result in significant morbidity and mortality and increased health care costs secondary to, among other things, increased rehospitalization rates.

## Immunonutrition within ERAS pathway

The issue of how best to implement immunonutrition within an ERAS framework remains unresolved. There are currently no studies that specifically address this issue with clear conclusions.

A recent study by Moya (2016) randomized patients into receiving standard oral nutrition versus immunonutrition within an ERAS pathway. Both groups were comparable in regard to age, sex, surgical risk, comorbidity, and analytical and nutritional parameters. The authors reported a decrease in the total number of complications in the immunonutrition group compared with the control group, primarily due to a significant decrease in infectious complications (23.8 vs. 10.7%, *P* < 0.0007). However, while the authors reported a potential benefit of immunonutrition within a colectomy ERAS with respect to surgical site infection (21/122 vs. 7/122), both of these rates are higher than the standard baseline for a laparoscopic colectomy population using oral and mechanical bowel preparation, bringing into question whether such a difference would be noticed in other institutions with lower baseline rates.

Another study, this one by Thorneblade (2017) and colleagues, supported the adoption of immune-enhancing nutrition before elective surgery as a way to reduce prolonged hospitalizations and improve the quality of surgical care. Three thousand three hundred seventy-five patients undergoing elective colorectal surgery were provided a preoperative checklist that recommended that patients take oral immunonutrition (237 mL, three times daily) for 5 days before elective colorectal resection. While the results showed there was an advantage in LOS (relative risk 0.77; 95% CI 0.58–1.01 *P* = 0.05), there was no evidence related to the reduction of specific or overall complications (relative risk 0.76; 95% CI 0.49–1.16). Furthermore, it must be understood that this was a non-controlled trial and also did not actually track compliance with the nutritional recommendations nor control the volume and constituents consumed.

Thus, while there have been studies on immunonutrition within an ERAS pathway, both studies have severe flaws in design and sample, bringing their overall conclusion into question.

## Conclusion

The topic of immunonutrition remains unresolved. Despite numerous metanalyses over the course of over 2 decades, there remains a major lack of well-conducted randomized trials in the literature.

Future research into this area should look to group patients based on preoperative risk adjustment using both image-guided assessment of sarcopenia and biomarker assessment of the nutritional and inflammatory state of populations of patients. Likely, candidates have CT measured sarcopenia scores, ultrasound assessment of rectus femoris, nutritional metabolic scores, systemic methylarginines, ornithine:citrulline ratio, and proline:citrulline ratio across the perioperative period in patients undergoing colorectal surgery within an Enhanced Recovery Program (ERP) program with a robust historical data set of specific outcomes. Only after this type of modern risk adjustment would the formulation of optimal nutrient formulas be accomplished. Lastly, with the optimal nutrition based on the stratification, we would be in a position to determine both the optimal timing and duration of immunotherapy within an ERP. Properly designed randomized control trials exploring these concepts are needed, however, before any conclusions can be definitively drawn.
